# The association between the systemic immune-inflammation index and in-hospital mortality among acute ischemic stroke with atrial fibrillation patients undergoing intravenous thrombolysis

**DOI:** 10.3389/fcvm.2025.1541762

**Published:** 2025-04-07

**Authors:** Kadiyan Aierken, Liang Ma, Yu Zhu, Xinyang Jin, Yajie Zhu, Jiahui Zhou, Jing Gao, Hongling Zhao, Tao Wang, Shijun Li

**Affiliations:** ^1^ Department of Cardiology, Central Hospital of Dalian University of Technology (Dalian Municipal Central Hospital), Dalian, China; ^2^ China Medical University, Shenyang, China; ^3^ Dalian Medical University, Dalian, China; ^4^ Department of Neurology, Central Hospital of Dalian University of Technology (Dalian Municipal Central Hospital), Dalian, China

**Keywords:** systemic immune-inflammation index, acute ischemic stroke, atrial fibrillation, intravenous thrombolysis, in-hospital mortality

## Abstract

**Objective:**

This study aimed to explore the relationship between the systemic immune-inflammatory index (SII) and the probability of in-hospital mortality among acute ischemic stroke (AIS) with atrial fibrillation (AF) patients undergoing intravenous thrombolysis.

**Methods:**

This single-center, retrospective observational study included individuals among AIS with AF who received intravenous thrombolysis. The SII is determined by taking the product of the platelet and neutrophil counts, followed by dividing this result by the lymphocyte count. In-hospital mortality was defined as a Modified Rankin Scale (mRS) score of 6 point. The investigation applied logistic regression models, along with subgroup, sensitivity, and receiver operating characteristic (ROC) curve analyses assessments, to explore the relationship between the SII and in-hospital mortality.

**Results:**

541 patients were included in this study, 50 (9.24%) of whom died during their hospital stay. Multifactorial logistic regression analyses using fully adjusted models, demonstrated that the SII is independently associated with the risk of in-hospital death. Patients with elevated SII levels experienced a significantly increased risk of in-hospital mortality, which was found to be 2.557 (95% CI: 1.154–5.665, *P* = 0.021) times greater compared to those with lower SII levels. Through multivariate logistic regression analyses, a notable correlation between the SII and the probability of death during hospitalization was observed across various subgroups, including individuals aged ≤75 and >75years, women, patients with persistent AF, those receiving thrombolytic therapy, diabetic and nondiabetic patients, individuals with BMI ≥24 kg/m^2^, and those with an admission National Institutes of Health Stroke Scale score ≤20 (*P* < 0.05). Two sensitivity analyses confirmed the robustness of this association from multiple perspectives (*P* < 0.05). ROC analysis demonstrated that the SII, the baseline model, and their combined model all showed strong predictive power for in-hospital mortality. Notably, the combined model outperformed the SII alone (*P* < 0.05). In addition, the predictive value of SII for in-hospital death was significantly higher than that of neutrophil-to-lymphocyte ratio (NLR) and platelet-to-lymphocyte ratio (PLR).

**Conclusion:**

A significant association has been observed between the risk of in-hospital death among AIS with AF individual undergoing intravenous thrombolysis and the SII.

## 1 Introduction

Atrial fibrillation (AF) represents one of the most frequently occurring cardiac arrhythmias worldwide. It is estimated that approximately 330 million individuals globally are affected by this condition, which has significant implications for both public health and clinical management (1, 2). The rising prevalence of AF, particularly among those over 80 years of age, where it exceeds 10% (3). As a result of AF, all-cause mortality can increase 1.5–3.5-fold, causing significant health consequences and economic burdens (4). Therefore, this has become a global public health issue. There is a high incidence of stroke throughout the world. In China, the lifetime risk of stroke is 39.9%, the highest worldwide, which indicates that approximately two out of every five people have a stroke during their lifetime (5). Approximately 70% of stroke cases are acute ischemic strokes (AIS), with management primarily targeting early reperfusion through intravenou thrombolysis, endovascular thrombectomy, or a combination of these methods (6). Although progress has been made in both technology and treatment approaches, Stroke continues to be a leading cause of both mortality and prolonged disability, presenting considerable challenges. AF**-**related strokes constitute over 79% of cardiac strokes, with risk correlating to AF burden (7). These strokes often result in worse clinical outcomes than non-AF strokes, including higher in-hospital mortality and poorer functional prognosis (8). Therefore, a straightforward, user-friendly and precise prognostic indicator is needed to enhance the prediction of clinical outcomes in AF patients undergoing intravenous thrombolysis for AIS.

Recent research indicates a significant link between inflammation and the outcomes of AF and AIS (9). Inflammatory disorders result in the migration of immune and inflammatory cells into the atrial tissues, resulting in the production of inflammatory mediators that harm cardiomyocytes and stimulate fibroblasts. This process induces inflammation and fibrosis by promoting collagen synthesis and releasing pro-inflammatory cytokines, thereby accelerating AF development (10). During AIS, the ischemic environment initiates a local immune response, and inflammatory cytokine production,leading to increased blood-brain barrier (BBB) permeability (11, 12). It is important to note that intravenous thrombolysis may alter leukocyte function and migration, promoting neutrophil degranulation, and consequently inducing BBB disruption. Vascular endothelial cell necrosis attracts leukocytes to the infarct site, while surface molecules on leukocytes promote neutrophil and macrophage infiltration, triggering acute inflammation (13, 14). Concurrently, BBB disruption causes necrotic neurons to activate microglia and release inflammatory mediators, intensifying the inflammatory cascade and resulting in severe brain damage and neurological deficits (12). Thus, inflammation likely influences clinical outcomes in AF patients undergoing intravenous thrombolysis for AIS and may be a key prognostic factor. The immune-inflammatory response is essential in the initiation and progression of a range of diseases. The assessment of inflammatory activity can be conducted through multiple hematological indices, which are calculated based on WBC and their subtypes. Extensive research has focused on the connection between blood count-derived inflammatory markers and clinical outcomes in AF and AIS (15–17). Inflammatory markers such as the monocytely-to-lymphocyte ratio (MLR), platelet-to-lymphocyte ratio (PLR), and neutrophil-to-lymphocyte ratio (NLR) have been suggested as prognostic indicators for AF and AIS, although findings have been inconsistent (16, 17). Due to the complex inflammation-related patho**-**physiological mechanisms in AF and AIS, relying solely on these blood count markers may limit their prognostic utility. Therefore, a more comprehensive indicator of inflammation is needed to assess its prognosis.

The systemic immune-inflammation index (SII) serves as a straightforward and consistent marker of inflammation, accurately reflecting immune responses at both local and systemic levels (18). The combined metric, which incorporates neutrophil, neutrophil, and platelet counts, offers more holistic assessment of the body's inflammatory state than isolated inflammatory markers. The SII, first investigated in 2014, has since emerged as a robust prognostic marker, particularly for patients with hepatocellular carcinoma (HCC) (19). Subsequent research has demonstrated that the SII provides superior predictive accuracy for long-term survival outcomes compared to other commonly used inflammatory markers, such as the pLR and NLR (20). A study conducted within the U.S. population identified a U-shaped correlation between the SII and mortality rates from both cardiovascular disease and cancer (21). Higher levels of the SII are linked to an increased risk of developing peripheral arterial disease (22), and atherosclerotic cardiovascular (23) conditions in American adults younger than 50 years. In AIS patients with severe internal carotid artery stenosis and stroke-associated pneumonia, elevated SII correlates with 120-day mortality (24). For AIS patients treated with standard-dose rt-pA intravenous thrombolysis, elevated SII is independently associated with poor prognosis (25). A study of 85,154 participants over 10 years found that higher SII levels increased the risk of stroke, its subtypes and mortality from all causes (26). Additionally, there is a notable positive association between the occurrence of paroxysmal AF and SII (27). A meta-analysis indicates that SII predicts poor functional outcomes in AIS patients (28).

In recent years, the SII has attracted growing attention. Its prognostic value is attributed to its comprehensive ability to capture inflammation and immune status compared to other markers SII, easily measurable from peripheral blood, is significantly linked to adverse outcomes after intravenous thrombolytic therapy in patients with AF and AIS. SII serves as a valuable tool for assessing the likelihood of in-hospital mortality in this patient population. The role of SII in assessing the risk of in-hospital mortality among patients with AF and AIS undergoing intravenous thrombolysis remains uncertain. This single-center retrospective cohort study thoroughly examined the connection between in-hospital mortality and SII among patients with AF and AIS treated with intravenous thrombolysis, providing new perspectives and a theoretical basis for improving treatment and prevention strategies in this population.

## 2 Materials and methods

### 2.1 Study population

Conducted at the Municipal Central Hospital of Dalian, this retrospective observational cohort study focused on individuals with AF and AIS who received intravenous thrombolysis between January 2018 and October 2023. The criteria for inclusion were defined as follows: (1) patients aged 18 years or older; (2) meeting the diagnostic criteria for AIS; (3) meeting AF diagnostic criteria, evidenced by a prior AF diagnosis or AF documentation on the initial or subsequent electrocardiogram during hospitalization; (4) receipt of intravenous thrombolytic therapy during hospitalization.The exclusion criteria included: (1) severe hepatic and renal failure; (2) malignant tumors; (3) life expectancy under one year; (4) severe psychiatric disorders hindering follow-up cooperation; (5) lack of parameters for SII assessment; (6) missing clinical information; (7) loss to follow-up. The study enrolled 541 patients, who were selected based on the use of well-defined inclusion and exclusion criteria. Relevant data were gathered from the medical records of patients who satisfied the inclusion criteria. The research protocol was authorized by the Institutional Review Board of the Municipal Central Hospital of Dalian, in accordance with the ethical principles outlined in the Declaration of Helsinki. Prior to enrollment, consent was obtained from all participants involved in the research.

### 2.2 Covariate collection and definition

Information regarding baseline attributes and participants' demographics was gathered, encompassing general information, vital signs, vascular risk factors, laboratory results, echocardiographic findings, and scores from relevant assessment tools. The initial records of these data were gathered for all patients.

Collected general information comprised age, time from onset to admission, decision-to-treatment (DNT) time, sex, height, weight, and body mass index (BMI). Measured vital signs included systolic blood pressure (SBP), diastolic blood pressure (DBP), and heart rate (HR). Vascular risk factors include AF type, stroke type, alcohol use, smoking status, diabetes mellitus, hypertension, history of stroke, congestive heart failure, and vascular disease, as specified below. Hypertension is diagnosed when a patient uses antihypertensive medications, has a systolic blood pressure of 140 mmHg or more, or exhibits a diastolic blood pressure of 90 mmHg or higher. Diabetes mellitus is diagnosed based on a preprandial fasting glucose level of 126 mg/dl or higher, postprandial glucose of 200 mg/dl or more, an HbA1c of 6.5% or greater. Alcohol consumption refers to any current intake, while smoking denotes any current tobacco use. Congestive heart failure is diagnosed based on clinical history. Vascular disease includes imaging-confirmed coronary artery disease, myocardial infarction, peripheral arterial disease (≥50% stenosis or revascularization), and aortic plaque. A history of stroke includes any prior occurrence of stroke, transient ischemic attack, or systemic embolism, encompassing both ischemic and hemorrhagic forms. Blood samples from the veins were collected from all patients within 24 h of admission, and the relevant laboratory measurements were subsequently recorded: triglycerides (TG), total cholesterol (TC), high-density lipoprotein cholesterol (HDL-C), low-density lipoprotein cholesterol (LDL-C), homocysteine (HCY), lipoprotein(a) [Lp(a)], uric acid (UA), creatinine (CREA), troponinT (cTnT), N-terminal Pro-B-type Natriuretic peptide (NT-proBNP), calcitonin (PCT), D-dimer, fibrinogen (FIB), lactate dehydrogenase (LDH), albumin (ALB), platelets (PLT), neutrophils, lymphocytes, monocytes, and hemoglobin (HGB). Echocardiographic parameters in this study were assessed by a specialized sonographer, specifically left atrial diameter (LAD), left ventricular diameter (LVD), right atrial diameter (RAD), right ventricular diameter (RVD), left ventricular ejection fraction (LVEF), and fractional shortening (FS), were evaluated by a specialized sonographer.

We classified AF based on the timing of episodes, specifically distinguishing between paroxysmal and persistent AF, as evaluated by specialized cardiologists. The etiology of AIS was determined using the TOAST classification system, which categorizes AIS into atherosclerotic large artery, cardiogenic embolism, small artery occlusion, other causes, and unknown causes, as assessed by a specialized neurologist. Hemorrhagic transformation (HT) was defined by the presence or absence of bleeding on head CT/MRI 24 h after intravenous thrombolytic therapy. The CHA2DS2-VASc-60 score is a tool used to assess stroke risk in patients with Asian AF. Each letter represents a specific clinical factor and each factor is assigned a different score as follows: C: Heart failure (congestive heart failure) or left ventricular insufficiency (1 score); H: Hypertension (1 score); A2: Age ≥65 years (2 score); D: Diabetes (1 score); S2: History of stroke, TIA, or thromboembolism (2 score); V: History of vascular disease, including coronary heart disease, peripheral arterial disease, or aortic dissection (1 score); A: Age (60–64years, 1 score); Sc: Female (1 score). Additionally, we collected National Institutes of Health Stroke Scale (NIHSS), Essen Stroke Risk Score (ESRS), and modified Rankin Scale (mRS). The NIHSS is a standardized tool for assessing the severity and prognosis of stroke. The NIHSS scoring system consists of 11 items, each of which is assigned a score based on the severity of the patient's condition, with a maximum total of 42 points. Scores were classified into mild to moderate (NIHSS ≤20) and severe (NIHSS >20). The ESRS incorporates a range of clinical factors, assigning up to 9 points based on the presence of specific cardiovascular and metabolic conditions. The mRS is a tool used to assess functional recovery and quality of life in patients who have had a stroke or other neurological condition. Scores ranged from 0 (no disability) to 6 (death), with each level having clear definitions that were easy to operationalize and understand clinically.

### 2.3 Inflammatory index and the assessment of in-hospital mortality

In this research, the exposure variable was represented by the SII, which was computed using the formula: SII = platelet count × neutrophil count/lymphocyte count. Based on the median SII value, participants were divided into two groups: low SII (SII ≤ 806.15, *n* = 270) and high SII (SII > 806.15, *n* = 271). Neutrophil-to-lymphocyte ratio (NLR) was the ratio of the neutrophil count to the lymphocyte count, and platelet-to-lymphocyte ratio (PLR) was the ratio of platelet to lymphocyte count.

An mRS score of 6 point at discharge was defined as death, establishing in-hospital mortality as the primary endpoint of this study. The patients were categorized into two separate groups: in-hospital mortality (*n* = 491) and non-in-hospital mortality (*n* = 50).

### 2.4 Statistical analysis

For continuous variables that adhere to a normal distribution, the values are presented as the mean ± standard deviation. To compare the groups, differences are analyzed using the independent samples t-test, which evaluates the statistical significance of the mean differences between them. When continuous variables do not exhibit a normal distribution, the values are typically reported using the median, accompanied by the interquartile range to represent the spread of the data. To evaluate differences between groups, nonparametric statistical methods are utilized for analysis. For categorical variables, the results are presented in terms of frequencies and corresponding percentages. To assess differences between groups, the chi-square test is employed for statistical evaluation. Due to skewness, the SII was log-transformed (Log_10_SII) to validate its relationship with in-hospital mortality. Due to the relatively small Log_10_SII value, a 1-unit increase in this parameter led to a significant change in the risk of mortality. Therefore, it was necessary to expand the value by 10 times to normalize the effect value. One-way logistic regression was used to identify factors affecting in-hospital mortality. Variables that showed a *P*-value below 0.05 were selected for inclusion in the stepwise multifactorial logistic regression model to explore the independent association between SII and in-hospital mortality. Subgroup analyses were conducted to examine the associations between in-hospital mortality and SII within various categories, taking into account variables such as age, gender, type of AF, treatment approach, diabetes status, BMI, and the NIHSS score at admission. The relationship between the in-hospital mortality and SII remained strong, as demonstrated by sensitivity analyses that excluded patients with a prior stroke history and by categorizing based on both mean, and optimal SII cutoff values. The predictive and incremental value of SII for in-hospital mortality was evaluated using ROC curves alongside the baseline model, NLR and PLR. A *P*-value < 0.05 was regarded as statistically significant. Statistical analyses were performed with the use of SPSS version 29.0, R 4.3.4 and MedCalc version 22.001.

## 3 Results

### 3.1 Baseline characteristics

Table 1 indicated that patients who experienced in-hospital mortality were more likely to receive bridging therapy, had longer onset-to-admission times, a higher incidence of vascular disease, and faster heart rates compared to those who did not experience in-hospital mortality. The in-hospital mortality group showed increased HDL-C and decreased LDL-C levels, alongside significantly higher NT-proBNP, cTnT, PCT, D-dimer, LDH, leukocytes, neutrophils, SII, and LVD. In contrast, this group had a significantly lower LVEF. Furthermore, NIHSS scores at admission, ESRS, and pre-stroke CHA2DS2-VASc-60 scores were significantly higher (*P* < 0.05).

**Table 1:** Comparison of clinical characteristics between subgroups with and without in-hospital mortality.

Characteristics	Total population	In-hospital mortality	Non-in-hospital mortality	*P* value
*N*	541	50	491	
Age (years)	74.23 ± 10.46	75.42 ± 10.57	74.11 ± 19.46	0.398
Gender *n* (%)				0.256
Female	219.0 (40.5%)	24.0 (48.0%)	195.0 (39.7%)	
Male	322.0 (59.5%)	26.0 (52.0%)	296.0 (60.3%)	
Duration of atrial fibrillation (years)	3.00 (0.62, 10.00)	12.0 (6.00, 21.25)	2.00 (0.21, 6.50)	0.091
Types of atrial fibrillation *n* (%)				0.091
Paroxysmal atrial fibrillation	223.0 (41.2%)	15.0 (30.0%)	208.0 (42.4%)	
Persistent atrial fibrillation	318.0 (58.8%)	35.0 (70.0%)	283.0 (57.6%)	
TOAST *n* (%)				0.066
Cardioembolism	326.0 (60.3%)	30.0 (60.0%)	296.0 (60.3%)	
Large**-**artery atherosclerosis	125.0 (23.1%)	8.0 (16.0%)	117.0 (23.8%)	
Small**-**vessel occlusion	57.0 (10.5%)	6.0 (12.0%)	51.0 (10.4%)	
Other determined etiology	18.0 (3.3%)	5.0 (10.0%)	13.0 (2.6%)	
Undetermined etiology	15.0 (2.8%)	1.0 (2.0%)	14.0 (2.9%)	
Therapeutic Modaliyies *n* (%)				0.005
Thrombolytic therapy	412.0 (76.2%)	30.0 (60.0%)	382.0 (77.8%)	
Bridging therapy	129.0 (23.8%)	20.0 (40.0%)	109.0 (22.2%)	
Onset**-**to**-**door (hours)	1.58 (1.00, 3.00)	2.00 (1.00, 3.25)	1.58 (1.00, 3.00)	0.035
DNT (minutes)	30.00 (26.00, 36.00)	30.00 (28.00, 38.25)	30.00 (26.00, 36.00)	0.204
Smoking *n* (%)	201.0 (37.2%)	18.0 (36.0%)	183.0 (37.3%)	0.859
Drinking *n* (%)	166.0 (30.7%)	16.0 (32.0%)	150.0 (30.5%)	0.832
Hypertension *n* (%)	377.0 (69.7%)	37.0 (74.0%)	340.0 (69.2%)	0.486
Diabetes *n* (%)	145.0 (26.8%)	17.0 (34.0%)	128.0 (26.1%)	0.228
Congestive heart failure *n* (%)	76.0 (14.0%)	9.0 (18.0%)	67.0 (13.6%)	0.399
Prior stoke *n* (%)	93.0 (17.2%)	10.0 (20.0%)	83.0 (16.9%)	0.580
Vascular diseases *n* (%)	113.0 (20.9%)	16.0 (32.0%)	97.0 (19.8%)	0.042
HT *n* (%)	51.0 (9.43%)	7.0 (14.0%)	44 (9.0%)	0.245
BMI (kg/m^2^)	25.25 (22.86, 27.71)	22.55 (18.43, 28.21)	25.52 (23.06, 27.75)	0.715
SBP (mmHg)	156.00 (138.00, 170.00)	158.00 (142.25, 160.75)	155.50 (136.00, 174.50)	0.126
DBP (mmHg)	86.97 ± 13.72	90.30 ± 16.15	86.63 ± 13.42	0.072
HR (bpm)	84.00 (72.00, 102.00)	110.00 (92.50, 121.00)	80.00 (68.00, 98.00)	0.004
TC (mmol/L)	4.12 (3.39, 4.76)	4.04 (3.09, 5.21)	4.12 (3.41, 4.73)	0.419
TG (mmol/L)	1.01 (0.80, 1.45)	0.96 (0.56, 1.20)	1.01 (0.81, 1.50)	0.100
LDL**-**C (mmol/L)	2.85 ± 1.01	2.52 ± 1.13	2.88 ± 0.99	0.023
HDL**-**C (mmol/L)	1.07 (0.84, 1.19)	1.11 (0.73, 1.24)	1.06 (0.84, 1.20)	0.008
HCY (umol/L)	15.10 (12.08, 19.08)	16.45 (12.65, 20.05)	14.70 (12.03, 18.75)	0.523
Lp(a) (mg/L)	140.00 (100.00, 276.50)	150.00 (94.75, 204.00)	140.00 (101.50, 293.00)	0.387
UA (umol/L)	360.00 (292.75, 427.25)	420.00 (335.75, 428.50)	356.00 (289.00, 426.75)	0.717
CREA (umol/L)	77.50 (66.75, 93.75)	86.95 (76.75, 183.50)	75.90 (66.25, 92.25)	0.064
NT**-** proBNp (pg/ml)	2,084.63 (800.00, 4,675.00)	3,365.42 (1,927.89, 25,867.50)	1,852.43 (792.50, 4,606.25)	0.009
cTnT (ug/L)	0.02 (0.01, 0.03)	0.028 (0.02, 0.12)	0.02 (0.01, 0.03)	<0.001
PCT (ng/ml)	0.10 (0.05, 0.13)	0.12 (0.08, 1.89)	0.10 (0.04, 0.13)	0.031
D-dimer (ug/ml)	2.24 (1.09, 5.05)	3.55 (1.28, 9.51)	1.96 (1.00, 4.58)	0.015
FIB (g/L)	2.55 (2.16, 3.34)	4.09 (1.85, 5.22)	2.54 (2.17, 3.25)	0.558
LDH (U/L)	188.00 (168.75, 221.25)	203.00 (171.75, 277.25)	187.50 (168.25, 220.75)	<0.001
ALB (g/L)	39.20 (37.08, 41.53)	41.80 (39.15, 43.05)	38.75 (37.03, 41.40)	0.816
PLT (10^9^/L)	178.50 (149.75, 220.00)	170.00 (156.50, 265.00)	181.00 (149.25, 218.75)	0.918
WBC (10^9^/L)	7.07 (6.24, 8.75)	8.66 (6.58, 14.14)	7.06 (6.12, 8.71)	<0.001
Neutrophil count (10^9^/L)	4.99 (4.27, 6.94)	6.98 (3.68, 12.17)	4.96 (4.31, 6.84)	<0.001
Lymphocyte count (10^9^/L)	1.47 (1.17, 1.90)	1.47 (0.68, 2.17)	1.47 (1.18, 1.90)	0.002
Monocyte count (10^9^/L)	0.55 (0.40, 0.70)	0.65 (0.48, 1.07)	0.53 (0.37, 0.69)	0.059
HGB (g/L)	136.20 ± 18.27	136.62 ± 21.66	136.15 ± 17.92	0.864
SII (10^9^/L)	806.15 (462.10, 1,430.46)	1,036.14 (376.95, 2,863.43)	649.99 (411.93, 1,090.60)	<0.001
LAD (mm)	48.00 (44.75, 53.25)	50.50 (46.25, 56.75)	48.00 (44.25, 52.75)	0.549
RVD (mm)	21.50 (20.00, 24.00)	20.00 (18.75, 27.50)	22.00 (20.00, 24.00)	0.247
RAD (mm)	47.00 (45.00, 53.00)	51.00 (46.50, 55.25)	46.50 (44.25, 50.75)	0.300
LVD (mm)	50.50 (47.00, 54.00)	52.50 (43.00, 54.25)	50.00 (47.00, 53.75)	0.036
LVEF (%)	58.00 (54.75, 62.00)	50.00 (50.00, 56.00)	58.00 (55.00, 61.00)	<0.001
FS (%)	30.00 (28.00, 33.00)	29.50 (24.75, 33.00)	30.00 (28.25, 32.75)	0.246
Admission to NIHSS	6.5 (4.00, 10.00)	9.50 (6.25, 17.00)	6.00 (3.25, 9.75)	<0.001
ESRS	3.00 (3.00, 5.00)	5.00 (4.50, 5.25)	3.00 (3.00, 4.00)	<0.001
Pre**-**stroke CHA2DS2-VASC**-**60 score	3.81 ± 1.58	4.26 ± 1.63	3.76 ± 1.57	0.033

Abbreviation: SII, systemic immune-inflammatory index; HT, hemorrhagic transformation; DNT, decision-to-treatment time; BMI, body mass index; HR, heart rate; SBP, systolic blood pressure; DBP, diastolic blood pressure; TC, total cholesterol; TG, triglycerides; LDL**-**C, low-density lipoprotein cholesterol; HDL**-**C, high-density lipoprotein cholesterol; HCY, homocysteine; Lp(a), lipoprotein(a); UA, uric acid; CREA, creatinine; cTnT, troponinT; NT-proBNP, N-terminal Pro-B-type Natriuretic peptide; pCT, calcitonin; FIB, fibrinogen; LDH, lactate dehydrogenase; ALB, albumin; pLT, platelets; WBC, leukocytes; HGB, hemoglobin; LAD, left atrial diameter; RVD, right ventricular diameter; RAD, right atrial diameter; LVD, left ventricular diameter; LVEF, left ventricular ejection fraction; FS, fractional shortening; NIHSS, National Institutes of Health Stroke Scale; ESRS, Essen Stroke Risk Score.

Table 2 indicated that the high SII group had increased in-hospital mortality, older age, a higher percentage of female patients, greater prevalence of persistent AF, more frequent use of bridging therapy, and elevated heart rates compared to the low SII group. Triglyceride levels decreased, whereas HDL-C levels increased. Additionally, levels of NT-proBNP, cTnT, D-dimer, FIB, and LDH were significantly elevated. platelet levels were higher, whereas hemoglobin levels were lower; LAD was decreased, RVD was increased, and LVEF were significantly lower. Furthermore, NIHSS scores at admission, ESRS, mRS scores and pre-stroke CHA2DS2**-**VASc-60 scores were significantly higher (*P* < 0.05).

**Table 2:** Clinical characteristics stratified by median SII.

Characteristic	Low SII group	High SII group	*P* value
*N*	270	271	
Age (years)	73.33 ± 10.19	75.12 ± 10.68	0.047
Gender *n* (%)			0.007
Female	94 (34.8%)	125 (46.1%)	
Male	176.0 (65.2%)	146 (53.9%)	
Duration of atrial fibrillation (years)	2.00 (0.00, 9.50)	3.00 (1.00, 10.00)	0.340
Types of atrial fibrillation *n* (%)			0.004
Paroxysmal atrial fibrillation	128 (47.4%)	95 (35.1%)	
Persistent atrial fibrillation	142 (52.6%)	176 (64.9%)	
TOAST *n* (%)			0.161
Cardioembolism	151 (55.9%)	175 (64.6%)	
Large-artery atherosclerosis	64 (23.7%)	61 (22.5%)	
Small-vessel occlusion	34 (12.6%)	23 (8.5%)	
Other determined etiology	12 (4.4%)	6 (2.2%)	
Undetermined etiology	9 (3.3%)	6 (2.2%)	
Therapeutic Modaliyies *n* (%)			<0.001
Thrombolytic therapy	232 (85.9%)	180 (66.4%)	
Bridging therapy	38 (14.1%)	91 (33.6%)	
Onset-to-door (hours)	1.50 (1.00, 2.75)	2.00 (1.00, 3.25)	0.286
DNT (minutes)	30.00 (24.50, 36.50)	30.00 (28.00, 36.00)	0.716
Smoking *n* (%)	111 (41.1%)	90 (33.2%)	0.057
Drinking *n* (%)	89 (33.0%)	77 (28.4%)	0.251
Hypertension *n* (%)	183 (67.8%)	194 (71.6%)	0.335
Diabetes *n* (%)	72 (26.7%)	73 (26.9%)	0.943
Congestive heart failure *n* (%)	32 (11.9%)	44 (16.2%)	0.142
Prior stoke *n* (%)	44 (16.3%)	49 (18.1%)	0.582
Vascular diseases *n* (%)	54 (20.0%)	59 (21.8%)	0.612
HT *n* (%)	10 (3.7%)	41 (15.1%)	<0.001
BMI (kg/m^2^)	25.06 (22.40, 27.92)	25.78 (23.77, 27.18)	0.290
SBP (mmHg)	155.00 (141.00, 170.50)	162.00 (126.00, 179.50)	0.646
DBP (mmHg)	87.54 ± 12.91	86.40 ± 14.49	0.335
HR (bpm)	80.00 (66.00, 95.00)	88.00 (72.00, 113.00)	<0.001
TC (mmol/L)	4.30 (3.48,5.00)	4.02 (3.15,4.71)	0.204
TG (mmol/L)	1.01 (0.83,1.49)	0.99 (0.73,1.43)	0.035
LDL**-**C (mmol/L)	2.81 ± 1.01	2.88 ± 1.01	0.438
HDL**-**C (mmol/L)	1.04 (0.83,1.28)	1.07 (0.87,1.17)	0.003
HCY (umol/L)	15.40 (12.45, 18.50)	14.40 (10.75, 19.35)	0.746
Lp(a) (mg/L)	197.00 (105.50, 352.50)	118.00 (99.00, 198.00)	0.209
UA (umol/L)	380.00 (294.50, 432.00)	328.00 (252.50, 396.00)	0.025
CREA (umol/L)	80.20 (68.30, 93.95)	74.30 (58.50, 95.50)	0.077
NT**-**proBNP (pg/ml)	1,500.00 (507.50, 3,000.00)	1,800.00 (838.00, 3,500.00)	0.027
cTnT (ug/L)	0.015 (0.011, 0.024)	0.020 (0.014, 0.038)	<0.001
PCT (ng/ml)	0.10 (0.04, 0.13)	0.10 (0.08, 0.13)	0.005
D-dimer (ug/ml)	1.58 (0.89, 3.68)	2.74 (1.16, 6.01)	<0.001
FIB (g/L)	2.74 (2.26, 3.26)	2.89 (2.38, 3.52)	0.014
LDH (U/L)	187.00 (164.50, 208.00)	215.00 (171.50, 229.50)	<0.001
ALB (g/L)	38.10 (37.05, 41.00)	41.20 (37.20, 43.00)	0.797
PLT (10^9^/L)	168.00 (128.50, 206.00)	215.00 (165.50, 282.50)	<0.001
WBC (10^9^/L)	6.54 (5.73, 7.77)	8.72 (7.52, 12.75)	<0.001
Neutrophil count (10^9^/L)	4.49 (3.24, 4.99)	7.13 (6.14, 10.38)	<0.001
Lymphocyte count (10^9^/L)	1.67 (1.31, 2.11)	1.30 (0.74, 1.60)	<0.001
Monocyte count (10^9^/L)	0.48 (0.34, 0.64)	0.66 (0.51, 0.92)	<0.001
HGB (g/L)	138.40 ± 16.17	134.01 ± 19.94	0.003
LAD (mm)	47.00 (44.50, 53.50)	49.00 (44.50, 53.50)	0.014
RVD (mm)	21.00 (20.00, 24.00)	22.00 (20.00, 25.00)	0.002
RAD (mm)	47.00 (45.00, 54.50)	47.00 (44.50, 50.00)	0.225
LVD (mm)	50.00 (47.00, 54.00)	51.00 (46.00, 53.00)	0.494
LVEF (%)	60.00 (55.00, 62.00)	55.00 (50.00, 60.00)	<0.001
FS (%)	30.00 (29.00, 32.00)	30.00 (25.50, 33.00)	0.606
Admission to NIHSS	5.00 (2.00, 8.00)	9.00 (6.00, 14.00)	<0.001
ESRS	3.00 (3.00, 5.00)	4.00 (3.00, 5.00)	<0.001
Pre**-**stroke CHA2DS2-VASC-60 score	3.66 ± 1.56	3.95 ± 1.60	0.036
In**-**hospital mortality *n* (%)	11.0 (4.1%)	39 (14.4%)	<0.001

Low SII: SII ≤ 806.15 (10^9^/L); High SII: SII > 806.15 (10^9^/L). Abbreviation: SII, systemic immune-inflammatory index, HT, hemorrhagic transformation; DNT, decision-to-treatment time; BMI, body mass index; HR, heart rate; SBP, systolic blood pressure; DBP, diastolic blood pressure; TC, total cholesterol; TG, triglycerides; LDL**-**C, low-density lipoprotein cholesterol; HDL**-**C, high-density lipoprotein cholesterol; HCY, homocysteine; Lp(a), lipoprotein(a); UA, uric acid; CREA, creatinine; cTnT, troponinT; NT-proBNP, N-terminal Pro-B-type Natriuretic peptide; PCT, calcitonin; FIB, fibrinogen; LDH, lactate dehydrogenase; ALB, albumin; pLT, platelets; WBC, leukocytes; HGB, hemoglobin; LAD, left atrial diameter; RVD, right ventricular diameter; RAD, right atrial diameter; LVD, left ventricular diameter; LVEF, left ventricular ejection fraction; FS, fractional shortening; NIHSS, National Institutes of Health Stroke Scale; ESRS, Essen Stroke Risk Score.

### 3.2 Correlation of SII with in-hospital mortality

Univariate logistic regression analysis showed a significant association between the risk of in-hospital mortality and various factors, as shown in Table 3. These included stroke type, treatment approach, vascular conditions, SBP, HR, LDL-C, HDL-C, CREA, NT-proBNP, D-dimer, FIB, leukocytes, neutrophils, monocytes, lymphocytes, SII, LVEF, NIHSS scores at admission, ESRS, mRS scores, and pre-stroke CHA2DS2-VASc-60 scores, all showing statistical significance (*P* < 0.05).

**Table 3:** Univariate logistic regression analysis of in-hospital mortality.

Characteristic	OR	95% CI	*P* value
Age	1.012	0.984–1.042	0.398
Male	0.714	0.398–1.279	0.257
Duration of atrial fibrillation	1.029	0.999–1.060	0.054
Persistent atrial fibrillation	1.715	0.913–3.223	0.094
TOAST			
Cardioembolism	Ref		
Large-artery atherosclerosis	0.675	0.301–1.515	0.340
Small-vessel occlusion	1.161	0.460–2.929	0.752
Other determined etiology	3.795	1.266–11.374	0.017
Undetermined etiology	0.705	0.090–5.547	0.740
Therapeutic Modaliyies			
Thrombolytic therapy	Ref		
Bridging therapy	2.336	1.276–4.276	0.006
Onset-to-door	0.848	0.698–1.029	0.094
DNT	1.007	0.986–1.028	0.505
Smoking	0.947	0.517–1.735	0.859
Drinking	1.070	0.573–1.998	0.832
Hypertension	1.264	0.653–2.446	0.487
Diabetes	1.461	0.787–2.713	0.230
Congestive heart failure	1.389	0.646–2.989	0.400
Prior stoke	1.229	0.591–2.555	0.581
Vascular diseases	1.911	1.014–3.605	0.045
HT	1.654	0.702–3.896	0.250
BMI	0.997	0.922–1.078	0.938
SBP	1.014	1.001–1.027	0.031
DBP	1.020	0.998–1.042	0.072
HR	1.019	1.007–1.030	0.002
TC	0.960	0.709–1.300	0.793
TG	0.868	0.551–1.369	0.543
LDL**-**C	0.692	0.502–0.953	0.024
HDL**-**C	3.661	1.490–8.997	0.005
HCY	0.990	0.954–1.028	0.618
Lp(a)	0.999	0.997–1.001	0.225
UA	1.001	0.998–1.004	0.417
CREA	1.005	1.001–1.010	0.029
NT**-**proBNP	1.000	1.000–1.000	<0.001
cTnT	0.910	0.451–1.835	0.792
PCT	1.012	0.893–1.148	0.848
D-dimer	1.027	1.009–1.046	0.004
FIB	1.279	1.004–1.628	0.046
LDH	1.001	1.000–1.003	0.111
ALB	1.014	0.950–1.082	0.682
PLT	1.000	0.996–1.005	0.852
WBC	1.212	1.129–1.301	<0.001
Neutrophil count	1.282	1.188–1.383	<0.001
Lymphocyte count	0.507	0.295–0.870	0.014
Monocyte count	4.898	1.823–13.156	0.002
HGB	1.001	0.986–1.018	0.864
SII	1.000	1.000–1.001	<0.001
Log_10_SII*10	1.283	1.174–1.402	<0.001
LAD	0.975	0.930–1.022	0.284
RVD	1.040	0.926–1.168	0.508
RAD	1.021	0.943–1.105	0.609
LVD	1.066	0.997–1.140	0.061
LVEF	0.922	0.888–0.957	<0.001
FS	0.936	0.834–1.051	0.264
Admission to NIHSS	1.100	1.067–1.134	<0.001
ESRS	2.544	1.915–3.381	<0.001
Pre**-**stroke CHA2DS2-VASC-60 score	1.220	1.015–1.467	0.034

Abbreviation: SII, systemic immune-inflammatory index, HT, hemorrhagic transformation; DNT, decision-to-treatment time; BMI, body mass index; HR, heart rate; SBP, systolic blood pressure; DBP, diastolic blood pressure; TC, total cholesterol; TG, triglycerides; LDL**-**C, low-density lipoprotein cholesterol; HDL**-**C, high-density lipoprotein cholesterol; HCY, homocysteine; Lp(a), lipoprotein(a); UA, uric acid; CREA, creatinine; cTnT, troponinT; NT-proBNP, N-terminal Pro-B-type Natriuretic peptide; PCT, calcitonin; FIB, fibrinogen; LDH, lactate dehydrogenase; ALB, albumin; pLT, platelets; WBC, leukocytes; HGB, hemoglobin; LAD, left atrial diameter; RVD, right ventricular diameter; RAD, right atrial diameter; LVD, left ventricular diameter; LVEF, left ventricular ejection fraction; FS, fractional shortening; NIHSS, National Institutes of Health Stroke Scale; ESRS, Essen Stroke Risk Score.

The relationship between SII and in-hospital deaths after adjusting for potential covariatesare shown in Table 4. In Model 1, without adjustmented, the SII as a continuous variable correlated with in-hospital mortality risk. The classification of SII demonstrated that patients in the high SII group faced a 3.958 (95% CI: 1.981–7.908, *P* < 0.001) times greater risk of in-hospital mortality compared to those in the low SII group. In Model 2, after adjusting for stroke type, treatment modality, and vascular disease, SII was significantly linked to in-hospital mortality risk, with each 1**-**unit increase in SII raising the risk by 0.1% and each Log_10_SII*10 increase by 31.5% (*P* < 0.001). Patients with a high SII faced a significantly increased risk of in-hospital mortality, with a 3.985**-**fold rise (95% CI: 1.912–8.304, *P* < 0.001) compared to those with a low SII. After adjusting for SBP, HR, LDL**-**C, HDL**-**C, CREA, FIB, D**-**dimer, NT**-**proBNP, LVEF, NIHSS scores at admission, ESRS, mRS scores, and pre-stroke CHA2DS2-VASc-60 scores in Model 2, multivariate logistic regression analysis confirmed SII's independent association with in-hospital mortality risk, patients in the high SII category exhibited a 2.557 (95% CI: 1.154–5.665, *P* = 0.021) times higher likelihood of in-hospital mortality compared to those in the low SII category.

**Table 4:** Multivariable logistic regression analysis of SII and in-hospital mortality.

Characteristic	Model 1			Model 2			Model 3		
	OR	95% CI	*P* value	OR	95% CI	*P* value	OR	95% CI	*P* value
SII	1.000	1.000–1.001	<0.001	1.001	1.000–1.001	<0.001	1.000	1.000–1.001	<0.001
Log_10_SII*10	1.283	1.174–1.402	<0.001	1.315	1.198–1.444	<0.001	1.246	1.118–1.390	<0.001
SII									
Low SII	Ref			Ref			Ref		
High SII	3.958	1.981–7.908	<0.001	3.985	1.912–8.304	<0.001	2.557	1.154–5.665	0.021

Model 1: unadjusted. Model 2: adjusting for stroke type, therapeutic modaliyies and vascular disease. Model 3: adjusting for stroke type, therapeutic modaliyies, vascular disease, SBP, HR, LDL**-**C, HDL**-**C, CREA, FIB, D**-**dimer, NT**-**proBNP, LVEF, admission to NIHSS, ESRS, and pre**-**stroke CHA2DS2-VASC**-**60 score. SII, systemic immune-inflammatory index, HR, heart rate; SBP, systolic blood pressure; LDL**-**C, low-density lipoprotein cholesterol; HDL**-**C, high-density lipoprotein cholesterol; CREA, creatinine; NT-proBNP, N-terminal Pro-B-type Natriuretic peptide; FIB, fibrinogen; LVEF, left ventricular ejection fraction; NIHSS, National Institutes of Health Stroke Scale; ESRS, Essen Stroke Risk Score.

### 3.3 Subgroup analysis

**Figure 1:**
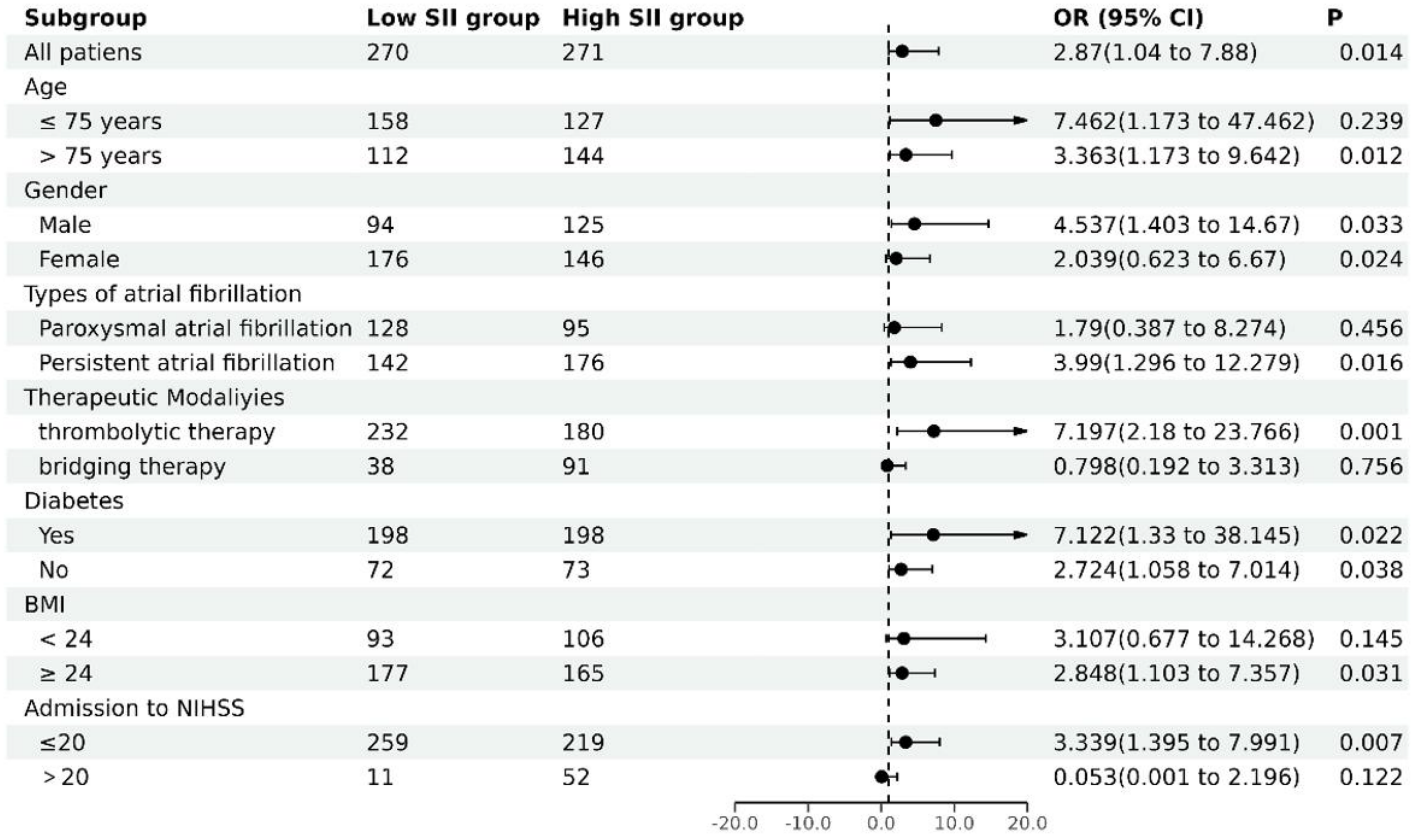
Subgroup analysis of multifactorial stratified associations of SII with in-hospital mortality.

To further explore the relationship between SII and in-hospital mortality in different subgroups, this study conducted subgroup analyses based on age, gender, type of AF, treatment modality, diabetes, BMI, and admission to NIHSS scores. According to Table 5 and Figure 1, after adjusting for various factors such as stroke type, treatment modality, stroke type, therapeutic modaliyies, vascular disease, SBP, HR, LDL**-**C, HDL**-**C, CREA, FIB, D**-**dimer, NT**-**proBNP, LVEF, admission to NIHSS, ESRS, and pre**-**stroke CHA2DS2**-**VASC**-**60 score, a significant association exists between the SII and in-hospital mortality risk. Specifically, multivariate logistic regression analyses indicate that patients with high SII have a significantly increased risk of in-hospital mortality compared to those with low SII, with odds ratios of 7.462 (95% CI: 1.173–47.462, *P* = 0.033) for patients aged ≤75 years and 3.363 (95% CI: 1.173–9.642, *P* = 0.024) for those aged >75 years. Among female patients, those with a high SII had a 4.537**-**fold (95% CI: 1.403–14.670, *P* = 0.012) higher risk of in-hospital mortality compared to those with a low SII. Among individuals with persistent AF, those classified in the high SII category exhibited a 3.990**-**fold (95% CI: 1.296–12.279, *P* = 0.016) greater likelihood of in-hospital mortality compared to patients in the low SII category. In patients undergoing thrombolytic therapy, those in the high SII group had a 7.197**-**fold (95% CI: 2.180–23.766, *P* = 0.001) increased risk of in-hospital mortality compared to the low SII group. In patients with and without diabetes, the group with elevated SII demonstrated a markedly higher risk of in-hospital mortality, with odds ratios of 7.122 (95% CI: 1.330–38.145, *P* = 0.022) for diabetics and 2.724 (95% CI: 1.058–7.014, *P* = 0.038) for non-diabetics. Additionally, among patients with a BMI ≥24 kg/m^2^, the high SII group had a 2.848**-**fold (95% CI: 1.103–7.357, *P* = 0.031) higher risk of in-hospital mortality compared to the low SII group. Patients who had an NIHSS score of 20 or less and were placed in the high SII category faced a 3.339**-**fold (95% CI: 1.395–7.991, *P* = 0.007) increased risk of in-hospital mortality compared to those in the low SII group.

**Table 5:** Stratified associations between SII and in-hospital mortality.

Subgroups	High SII group vs. low SII group		
	OR	95% CI	*P* value
Age			
≤75 years	7.462	1.173–47.462	0.033
>75 years	3.363	1.173–9.642	0.024
Gender			
Female	2.039	0.623–6.670	0.239
Male	4.537	1.403–14.670	0.012
Types of atrial fibrillation			
Paroxysmal atrial fibrillation	1.790	0.387–8.274	0.456
Persistent atrial fibrillation	3.990	1.296–12.279	0.016
Therapeutic modaliyies			
Thrombolytic therapy	7.197	2.180–23.766	0.001
Bridging therapy	0.798	0.192–3.313	0.756
Diabetes			
Yes	7.122	1.330–38.145	0.022
No	2.724	1.058–7.014	0.038
BMI			
<24 kg/m^2^	3.107	0.677–14.268	0.145
≥24 kg/m^2^	2.848	1.103–7.357	0.031
Admission to NIHSS			
≤20	3.339	1.395–7.991	0.007
>20	0.053	0.001–2.196	0.122

Subgroup analysis adjusted for stroke type, therapeutic modaliyies, vascular disease, systolic blood pressure, heart rate, low-density lipoprotein cholesterol, high-density lipoprotein cholesterol, creatinine, fibrinogen, D**-**dimer, N-terminal Pro-B-type Natriuretic peptide, left ventricular ejection fraction, admission to NIHSS, Essen Stroke Risk Score, and pre**-**stroke CHA2DS2**-**VASC**-**60 score. Abbreviation: SII, systemic immune-inflammatory index, NIHSS, National Institutes of Health Stroke Scale.

### 3.4 Sensitivity analysis

As shown in Table 6, this association remained significant regardless of whether SII was analyzed as a continuous or categorical variable. Across all three models, the risk of in-hospital mortality rose with each 1-unit increment in Log_10_SII*10: Model 1 (unadjusted) showed a 1.307 **-**fold (*P* < 0.001) increase; Model 2 (adjusted for stroke type, treatment modality, and vascular disease) showed a 1.351-fold (*P* < 0.001) increase; and Model 3 (fully adjusted) showed an 1.332 **-** fold (*P* < 0.001) increase. Patients with high SII had a notably higher risk of in-hospital mortality compared to those with low SII, the odds ratios were 4.589, 5.475, and 4.334 (*P* < 0.001).

**Table 6:** Multivariable logistic regression analysis of SII and in-hospital mortality: exclusion of patients with a prior stroke.

Variables	Model 1			Model 2			Model 3		
	OR	95% CI	*P* value	OR	95% CI	*P* value	OR	95% CI	*P* value
SII	1.000	1.000–1.001	<0.001	1.001	1.000–1.001	<0.001	1.001	1.000–1.001	<0.001
Log_10_SII*10	1.307	1.183–1.444	<0.001	1.351	1.214–1.503	<0.001	1.332	1.176–1.509	<0.001
SII									
Low SII	Ref			Ref			Ref		
High SII	4.589	2.065–10.202	<0.001	5.475	2.357–12.716	<0.001	4.334	1.818–10.331	<0.001

Model 1: unadjusted. Model 2: adjusting for stroke type, therapeutic modaliyies and vascular disease. Model3: adjusting for stroke type, therapeutic modaliyies, vascular disease, SBP, HR, LDL**-**C, HDL**-**C, CREA, FIB, D-dimer, NT**-**proBNP, LVEF, admission to NIHSS, ESRS, and pre**-**stroke CHA2DS2**-**VASC**-**60 score. Abbreviation: SII, systemic immune-inflammatory index, HR, heart rate; SBP, systolic blood pressure; LDL**-**C,low-density lipoprotein cholesterol; HDL**-**C, high-density lipoprotein cholesterol; CREA, creatinine; NT-proBNP, N-terminal Pro-B-type Natriuretic peptide; FIB, fibrinogen; LVEF, left ventricular ejection fraction; NIHSS, National Institutes of Health Stroke Scale; ESRS, Essen Stroke Risk Score.

Table 7 showed that, in the total population, unadjusted model 1 logistic regression analysis revealed that stratifying patients by the mean optimal SII cutoff value resulted in the high SII group having a 3.893–4.479 times greater risk of in-hospital mortality compared to the low SII group when SII was a categorical variable (*P* < 0.001). In model 2, accounting for stroke type, treatment modality, and vascular disease, the high SII group had a significantly increased risk of in-hospital mortality compared to the low SII group, with odds ratios of 4.270 and 5.314 (*P* < 0.001). Model 2, adjusted for SBP, HR, LDL-C, HDL-C, CREA, FIB, D-dimer, NT-proBNP, LVEF, NIHSS scores at admission, ESRS, mRS scores and pre-stroke CHA2DS2-VASc-60 scores, confirmed a significant independent association between the risk of in-hospital mortality and the high SII group. The high SII group exhibited a significantly increased risk of in-hospital mortality compared to the low SII group, with odds ratios of 3.146 and 3.436 (*P* = 0.001). In patients without a stroke history, unadjusted logistic regression analysis (model 1) showed that categorizing SII based on its mean optimal cutoff revealed a higher mortality risk in the high SII group, with odds ratios of 4.457 and 5.160 (*P* < 0.001). In model 2, after adjusting for stroke type, treatment modality, and vascular disease, the high SII group had a significantly increased risk of in-hospital mortality compared to the low SII group, with odds ratios of 5.159 and 6.063 (*P* < 0.001). Model 2, adjusted for SBP, HR, LDL-C, HDL-C, CREA, FIB, D-dimer, NT**-**proBNP, LVEF, NIHSS scores at admission, ESRS, mRS scores, and pre**-**stroke CHA2DS2**-**VASc**-**60 scores, confirmed the independent association between the high SII group and the risk of in-hospital mortality, and in-hospital mortality was 5.330 and 5.751 (*P* < 0.001) times higher in the high SII group.

**Table 7:** Multivariable logistic regression analysis of SII and in-hospital mortality: grouped by different SII thresholds.

Variables	Model 1			Model 2			Model 3		
	OR	95% CI	*P* value	OR	95% CI	*P* value	OR	95% CI	*P* value
In the total population									
Average SII									
Low SII	Ref			Ref			Ref		
High SII	3.893	2.130–7.116	<0.001	4.270	2.294–7.946	<0.001	3.146	1.617–6.119	0.001
SII optimal cut-off value									
Low SII	Ref			Ref			Ref		
High SII	4.749	2.460–9.168	<0.001	5.314	2.687–10.511	<0.001	3.436	1.659–7.114	0.001
In the population without a history of stroke									
Average SII									
Low SII	Ref			Ref			Ref		
High SII	4.457	2.250–8.831	<0.001	5.159	2.505–10.626	<0.001	5.330	2.458–11.557	<0.001
SII optimal cut-off value									
Low SII	Ref			Ref			Ref		
High SII	5.160	2.453–10.852	<0.001	6.063	2.754–13.352	<0.001	5.751	2.541–13.016	<0.001

Model 1: unadjusted. Model 2: Adjusting for stroke type,therapeutic modaliyies, and vascular disease. Model3: Adjusting for stroke type, therapeutic modaliyies, vascular disease, SBP, HR, LDL**-**C, HDL**-**C, CREA, FIB, D-dimer, NT**-**proBNP, LVEF, admission to NIHSS, ESRS, and pre**-**stroke CHA2DS2-VASC**-**60 score. Abbreviation: SII, systemic immune-inflammatory index, HR, heart rate; SBP, systolic blood pressure; LDL**-**C, low-density lipoprotein cholesterol; HDL**-**C, high-density lipoprotein cholesterol; CREA, creatinine; NT-proBNP, N-terminal Pro-B-type Natriuretic peptide; FIB, fibrinogen; LVEF, left ventricular ejection fraction; NIHSS, National Institutes of Health Stroke Scale; ESRS, Essen Stroke Risk Score.

### 3.5 ROC analysis

**Figure 2:**
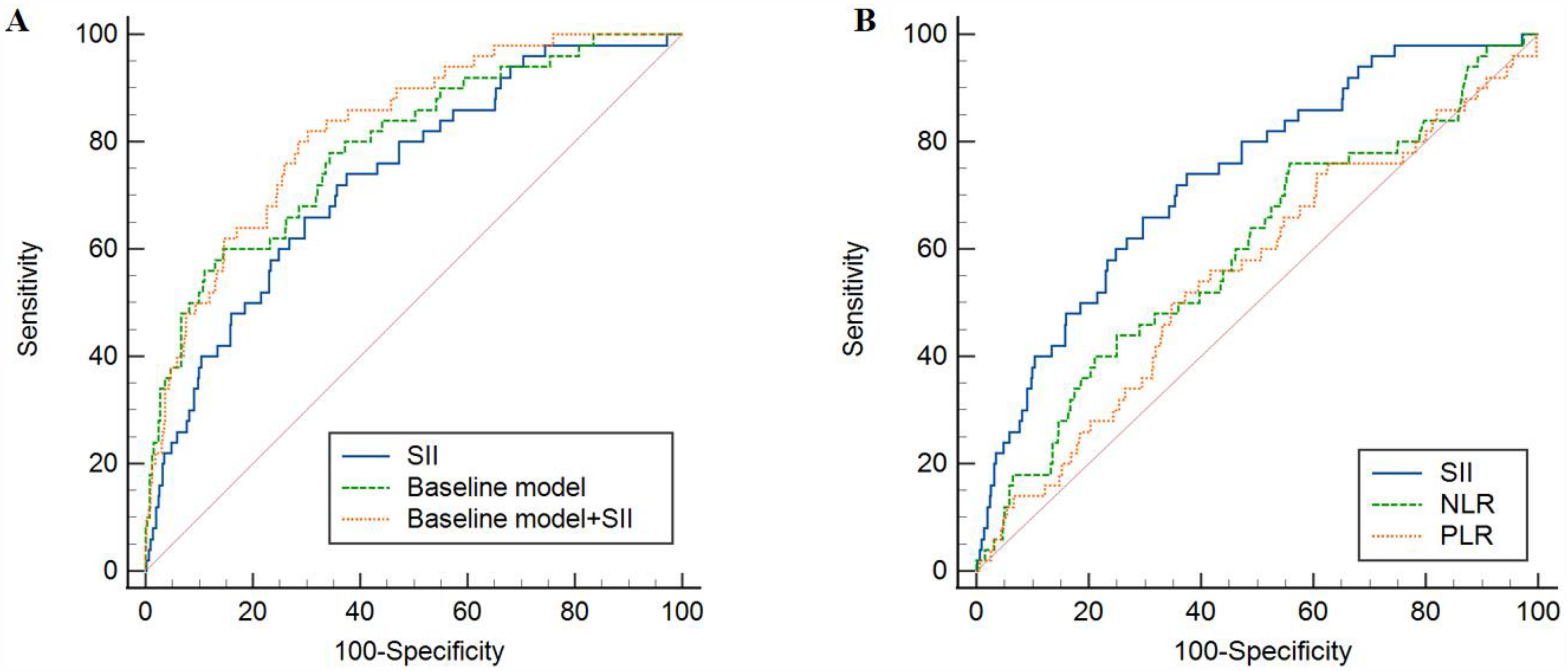
ROC analysis of the predictive value of SII, NLR, PLR and baseline models for in-hospital mortality. **(A)** Comparison between SII and baseline model; **(B)** Comparison between SII and NLR, PLR. SII, systemic immune-inflammatory index; NLR, neutrophil-to-lymphocyte ratio; PLR, platelet-to-lymphocyte ratio; ROC, receiver operating characteristic.

The ROC analysis, as shown in Table 8 and Figure 2, highlighted the strong predictive value of the SII, the baseline model, and their combination for predicting in-hospital mortality (*P* < 0.001). Notably, the combination of SII and the baseline model outperformed SII alone in terms of predictive ability (*P* < 0.05). Further ROC curve analysis of NLR and PLR showed that SII had a higher predictive value for in-hospital mortality than NLR and PLR (*P* < 0.05).

**Table 8:** ROC analysis of the predictive value of SII and baseline models for in-hospital mortality.

Variables	AUC	95% CI	*P* value	*P* for comparison
SII	0.734	0.663–0.805	<0.001	Ref
Baseline model	0.790	0.722–0.859	<0.001	0.273
SII + Baseline model	0.822	0.765–0.879	<0.001	0.012
NLR	0.597	0.554–0.638	0.028	0.022
PLR	0.557	0.514–0.599	0.199	0.002

Baseline models included stroke type, therapeutic modaliyies, vascular disease, SBP, HR, LDL**-**C, HDL**-**C, CREA, FIB, D**-**dimer, NT-proBNP, LVEF, admission to NIHSS, ESRS, and pre**-**stroke CHA2DS2-VASC**-**60 score. Abbreviation: SII, systemic immune-inflammatory index, NLR, neutrophil-to-lymphocyte ratio; PLR, platelet-to-lymphocyte ratio; HR, heart rate; SBP, systolic blood pressure; LDL**-**C, low-density lipoprotein cholesterol; HDL**-**C, high-density lipoprotein cholesterol; CREA, creatinine; NT-proBNP, N-terminal Pro-B-type Natriuretic peptide; FIB, fibrinogen; LVEF, left ventricular ejection fraction; NIHSS, National Institutes of Health Stroke Scale; ESRS, Essen Stroke Risk Score.

## 4 Discussion

As far as we are aware, this was the first study to explore the link between the SII and in-hospital mortality among AIS with AF patients undergoing intravenous thrombolysis. Our findings demonstrated a robust correlation between SII levels and the risk of in-hospital mortality in this patient cohort, particularly in those without a prior stroke history, with higher SII levels linked to increased mortality risk, both as continuous and categorical variables. In the fully adjusted model, a 1-unit rise in log_10_SII*10 was linked to an 1.332-fold increase in the risk of in-hospital mortality. Additionally, the high SII group exhibited a 4.334 times greater risk of in**-**hospital mortality compared to the low SII group. The analysis using the ROC curve demonstrated that the SII, the baseline model, and their combination all had strong predictive value for in-hospital mortality. Moreover, the combined model demonstrated superior predictive ability compared to the SII alone. Therefore, SII could be a crucial risk factor for AF patients who have suffered an AIS. Additionally, it could prove to be an important resource for healthcare professionals when making clinical decisions.

Recent research has investigated the connection between inflammation indicators and the outcomes of cardiovascular and cerebrovascular diseases, highlighting the SII serves as a prognostic indicator of condition severity and fatality in various conditions, including cancer, heart failure and AIS disorders (29–32). For example, a study involving 578 elderly patients with acute myocardial infarction has shown that the SII was not only independently associated with the occurrence of the no-reflow phenomenon after percutaneous coronary intervention in these patients, but also has a better predictive value for the no-reflow phenomenon than the NLR and the PLR (33). Besides, A previous study utilizing the Medical Information Mart for Intensive Care-IV (MIMIC-IV) database has identified a correlation between elevated SII levels and higher 30**-**day mortality rates in stroke patients (34). Meta-analyses confirm that elevated SII levels predict poor outcomes in ischemic stroke (35). Kaplan's research indicates that SII serves as a standalone predictor for AF recurrence and other studies have shown SII can predict new-onset AF post-myocardial infarction and mortality in severe AF cases (36–38). SII is also linked to prognosis in ischemic stroke and AF patients, and its association with AF in stroke patients is independent (39). This study further investigates SII's relationship with in-hospital mortality in AIS and AF patients undergoing intravenous thrombolysis, a topic not previously addressed in terms of in-hospital mortality risk or SII subgroup analysis. To assess the robustness of the correlation between the SII and in-hospital death rates, our study conducted a sensitivity analysis, categorizing patients according to different SII cutoff values, including the median, mean, and optimal thresholds. Earlier studies recognized the NIHSS score as an indicator of unfavorable outcomes in patients with AF and AIS (40). In this study, a subgroup analysis of admission NIHSS scores showed that patients with a score of ≤20 had a 3.339**-**fold higher risk of in-hospital mortality in the group with elevated SII levels relative to the group with reduced SII levels. Additionally, prior studies have linked higher SII with increased risks of diabetic nephropathy (41), and diabetic depression (42). In the subgroup of diabetic patients, individuals in the elevated SII group had a 7.122**-**old higher likelihood of in**-**hospital mortality compared to those in the low SII group. Additionally, we observed that in certain subgroups (e.g., male, diabetes), the association between SII and in-hospital mortality was more pronounced, whereas no statistically significant association was found in the “bridging therapy” subgroup. Regarding the potential mechanisms and heterogeneity underlying this phenomenon, we believe it may be related to several factors. First, the inflammatory response may manifest differently across populations. For example, male and diabetic patients may have higher baseline inflammation levels, which could enhance the predictive ability of SII in these subgroups. Second, patients receiving bridging therapy typically undergo a more standardized treatment process, including mechanical thrombectomy, which may reduce the independent predictive effect of SII on mortality risk. Additionally, the relatively small sample size of this subgroup may have also affected the statistical power.

The precise biological mechanisms that link inflammatory markers to intravenous thrombolysis and mortality in AF and AIS remain unclear. An alternative explanation for the link between inflammatory markers and both the development of AF and increased mortality could be considered is the elevated levels of neutrophils, lymphocytes, and platelets in peripheral blood. C-reactive protein (CRP), interleukins-2, interleukins-6, interleukins-8, interleukins-17, and monocyte chemotactic protein are inflammatory mediators capable of triggering the release of cytokines including tumor necrosis factor-alpha (TNF-α), thereby initiating an inflammatory cascade (43–46). Research indicates that individuals with AF display elevated concentrations of these markers compared to those without the condition (47–49). Basic research further highlights the role of inflammatory pathways in atrial electrophysiological and structural remodeling, which contributes to AF onset and progression (50, 51). Among AIS patients receiving intravenous thrombolytic therapy, neutrophils accumulate in the infarct core and penumbra due to blood-brain barrier disruption and increased cerebral edema (52). Neutrophils release inflammatory mediators that damage endothelial cells, and basement membranes, leading to further inflammatory molecule release and adverse outcomes in AIS patients undergoing thrombolytic therapy (53, 54). Inflammatory markers such as neutrophils, platelets, and lymphocytes may increase the risk of in-hospital mortality by promoting the onset and progression of intravenous thrombolysis in AF and AIS. The SII is involved in thrombosis, inflammation, and immune response. Elevated SII levels suggest thrombosis and immune dysregulation, linked to adverse outcomes and increased risk of complications and mortality (55). Moreover, the role of SII in AF and AIS is closely related to multiple pathophysiological processes, particularly thrombosis, endothelial dysfunction, and neuroinflammation. Firstly, SII reflects the overall state of immune response and inflammation in the body. High SII levels typically indicate a heightened inflammatory response and immune dysregulation, both of which play a crucial role in the onset and progression of AF and AIS (56, 57). In AF patients, chronic inflammation exacerbates thrombosis formation, especially when atrial endothelial function is impaired (58). Endothelial damage activates platelets, leading to thrombus formation and further contributing to intra-atrial thrombosis, thereby increasing the risk of stroke (59). Secondly, in AIS patients, thrombus formation is closely associated with endothelial dysfunction, as ischemia-induced reduced local blood flow damages vascular endothelial cells, triggering the accumulation of inflammatory cells like neutrophils and the release of cytokines, which exacerbate local inflammatory responses, a process particularly pronounced in patients with high inflammation levels, ultimately promoting thrombosis, worsening microvascular damage in the brain, and leading to more severe ischemic injury (60). Finally, the role of SII in neuroinflammation should not be overlooked, as in AIS patients undergoing thrombolytic therapy, the interplay between reperfusion injury and inflammatory responses exacerbates neuroinflammation, with high SII levels potentially sustaining inflammatory reactions by activating microglia and inducing neuronal apoptosis, thereby aggravating neurological damage and negatively impacting patient prognosis (61, 62). In conclusion, SII significantly influences the outcomes of AF and AIS patients through its interactions with thrombosis, endothelial dysfunction, and neuroinflammation.

## 5 Limitations

Although this study has yielded important findings, several limitations must be acknowledged. First, As this study was a single-center retrospective design, it may introduce selection bias. Moreover, the sample size was relatively small, especially for the mortality group with only 50 cases. This may affect the stability of the results. In the future, we will further expand the sample size and integrate multi-center data to enhance the universality of the research results. Second, SII was only evaluated within 24 h after admission, without tracking its dynamic changes during the treatment process. Inflammatory status is indeed likely to fluctuate with treatment. Therefore, combining dynamic monitoring will enable a more comprehensive understanding of the association between SII and prognosis. Additionally, the study's assessment of inflammatory status and its relation to in-hospital mortality risk was limited by the absence of key blood markers, including interleukins, ultrasensitive CRP and other inflammatory cytokines. Fourth, due to its r etrospective design, this study could not establish causality. Despite multivariate adjustments and subgroup analyses, clinical outcomes may have been influenced by unmeasured confounders. The study was exclusively designed to explore the connection between in-hospital mortality and SII in patients with AF and AIS who received intravenous thrombolysis. Further research is needed to explore the connection between long-term prognosis and SII in this cohort, as the evaluation of prolonged mortality risk was not incorporated.

## 6 Conclusion

The research identified a strong correlation between SII levels recorded within the first 24 h of admission and unfavorable clinical results, focusing specifically on the likelihood of in-hospital death among AIS with AF patients undergoing intravenous thrombolysis. Even after accounting for various confounding factors, the association remained significant, indicating that SII may serve as an important predictor of in-hospital mortality in this patient population. Keeping SII levels below a specific threshold might lower mortality risk. Future research should explore confounding factors influencing SII's impact on survival in among AIS with AF patients undergoing intravenous thrombolysis.

## Data Availability

The original contributions presented in the study are included in the article/Supplementary Material, further inquiries can be directed to the corresponding author.
